# An Approach for Rapid Assessment of Seismic Hazards in Turkey by Continuous GPS Data

**DOI:** 10.3390/s90100602

**Published:** 2009-01-20

**Authors:** Haluk Ozener, Asli Dogru, Ahmet Unlutepe

**Affiliations:** 1 Istanbul Technical University, Department of Geodesy and Photogrammetry Engineering, Surveying Technique Division, Maslak, 34469, Istanbul, Turkey; 2 Bogazici University, Kandilli Observatory and Earthquake Research Institute, Geodesy Department, Cengelkoy, 34680, Istanbul, Turkey; E-Mail: asli.dogru@boun.edu.tr; 3 Strabag Inc., Niagara Tunnel Facility Project, 2520 Stanley Avenue, Niagara Falls, ON L2E6S4, Canada; E-mail: ahunlu@yahoo.com

**Keywords:** GPS, deformation monitoring, strain analysis, seismic hazard, sensor web

## Abstract

The Earth is being monitored every day by all kinds of sensors. This leads an overflow of data in all branches of science nowadays, especially in Earth Sciences. Data storage and data processing are the problems to be solved by current technologies, as well as by those accessing and analyzing these large data sources. Once solutions have been created for collecting, storing and accessing data, then the challenge becomes how to effectively share data, applications and processing resources across many locations. The Global Positioning System (GPS) sensors are being used as geodetic instruments to precisely detect crustal motion in the Earth's surface. Rapid access to data provided by GPS sensors is becoming increasingly important for deformation monitoring and rapid hazard assessments. Today, reliable and fast collection and distribution of data is a challenge and advances in Internet technologies have made it easier to provide the needed data. This study describes a system which will be able to generate strain maps using data from continuous GPS stations for seismic hazard analysis. Strain rates are a key factor in seismic hazard analyses. Turkey is a country prone to earthquakes with a long history of seismic hazards and disasters. This situation has resulted in the studies by Earth scientists that focus on Turkey in order to improve their understanding of the Earth's crust structure and seismic hazards. Nevertheless, the construction of models, data access and analysis are often not fast as expected, but the combination of Internet technologies with continuous GPS sensors can be a solution to overcome this problem. This system would have the potential to answer many important questions to assess seismic hazards such as how much stretching, squashing and shearing is taking place in different parts of Turkey, and how do velocities change from place to place? Seismic hazard estimation is the most effective way to reduce earthquake losses. It is clear that reliability of data and on-line services will support the preparation of strategies for disaster management and planning to cope with hazards.

## 1. Introduction

Turkey is an earthquake-prone country has a long history of natural hazards and disasters. Approximately 96 percent of the land containing 66 percent of the active faults is affected by earthquake hazards and 98 percent of its population lives in these regions. The Marmara region includes 11 large cities with populations of more than one million and 75 percent of the country's largest industrial complexes. Scientific understanding of earthquakes is vital for assessing earthquake hazards, and earthquake hazard estimation is the most effective way for Earth scientists to reduce earthquake losses. Therefore the investigation of crustal strain, which means long-term prediction of earthquake hazards, can provide strategies for effective earthquake risk reduction.

In 1999, the Kocaeli earthquake which struck the densely populated industrial heartland of Turkey, was responsible for 20,000 deaths and 45,000 injuries and displaced more than 300,000 people. According to the UNDP (The United Nations Development Program) [1], Turkey is the third country in terms of deaths related to the earthquakes. Earthquake hazards cause an enormous cost to the society in terms of loss of life and property. It is clear that substantial savings could be made with better understanding of the events and improved prediction, which can help to mitigate the risks. In a narrow sense, an earthquake is a sudden failure process, but in a broad sense, it is a long-term complex stress accumulation and release process occurring in the Earth's crust. Therefore, scientific understanding of earthquakes is vital. As the population increases, urban development and construction works expand on areas susceptible to earthquakes. By means of a greater understanding of the causes and effects of earthquakes, it may be possible to reduce the damage and loss of life resulting from of these destructive phenomena.

This study describes a systematic approach to solving the problems related to converting data to information in earthquake research as quickly and effectively as possible. The rapid analysis of the huge amount of raw data gathered by the sensors that are increasing in number, especially in the scientific area of Space Geodesy, has gaining crucial importance for Earth scientists. In this case, if the needs for rapid analysis, interpretation and presentation are secured then the end results will ensure the high temporal resolution needed for accurate interpretation of earthquake phenomena, and this in turn should lead to mitigation of earthquake damage.

## 2. Continuous GPS Sensors

**Figure 1.**
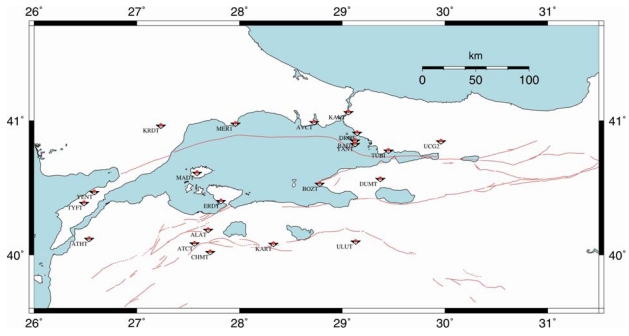
Location of GPS Sensors of MAGNET in Marmara Region (map produced by TUBITAK-MRC). Red lines indicate fault lines in the region.

Turkey has a GPS network named Marmara Continuous GPS Network (MAGNET) [2] collaborated by TUBITAK-MRC (Turkish Scientific and Technological Research Council – Marmara Research Center), MIT (Massachusetts Institute of Technology), ITU (Istanbul Technical University), GCM (General Command of Mapping) and Geodesy Department of KOERI (Kandilli Observatory and Earthquake Research Institute) of Bogazici University (Figure 1). The network currently has 21 permanent GPS stations operated by TUBITAK-MRC. GPS sensors are observing 24 hours at these points for deformation monitoring.

**Figure 2.**
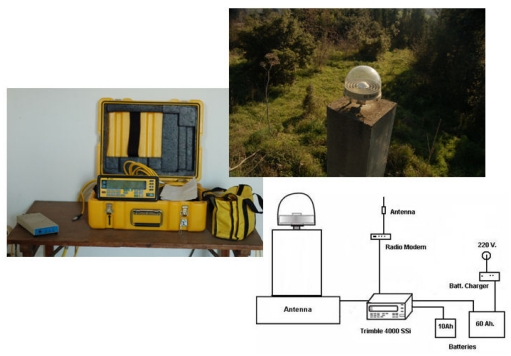
GPS sensor named KANT functioning 24 hours/day (Trimble 4000 SSE with a choke-ring model antenna).

One of these points, called KANT, is located at the Kandilli Campus of Bogazici University. KANT has been collecting data since July 6, 1999. GPS data is recorded 24 hours a day, with a logging interval of 30 seconds and the elevation mask is 10 degree. Figure 2 shows the GPS sensor (Trimble 4000 SSE with a choke-ring model antenna).

**Figure 3.**
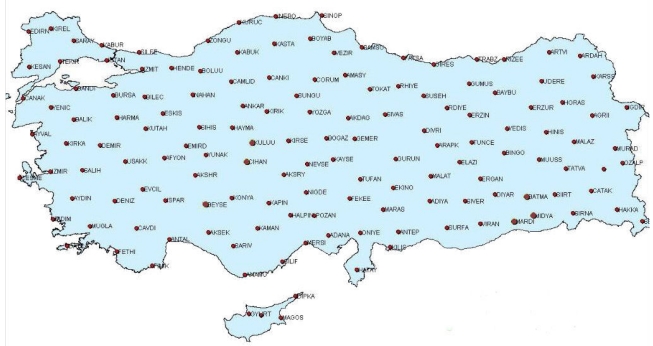
Locations of CORS-TR project GPS stations [3].

Unfortunately, it cannot be said that spatially dense GPS data are available for many regions of the country. In order to come up with a solution to the related shortcoming, a national project abbreviated as CORS-TR (Continuously Operating Reference Stations Project for Turkey) [3] has been initiated. Within the scope of the mentioned project, 150 continuously operating GPS stations are being installed. According to the project schedule, GPS sensors are to be located scattered over the entire terrain of the country with a station spacing of 80-90 km (Figure 3). Therefore, the data generated will provide significant challenges to tectonic studies in Turkey. This project is being conducted by Istanbul Kultur University.

**Figure 4.**
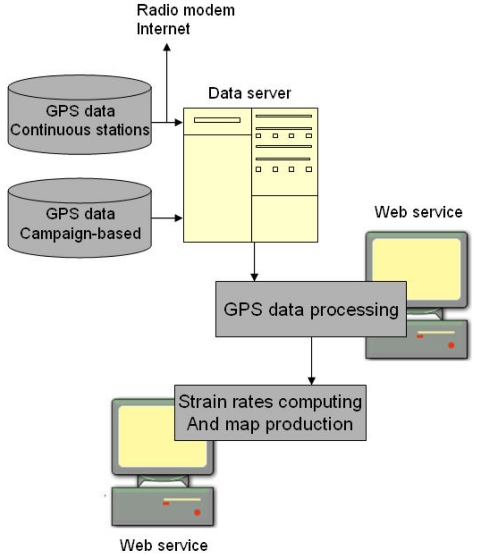
Data transfer and processing model.

Geodetic velocity products are continuously produced by processing the continuous data from the GPS sensors. However, an automatic GPS processor is required to provide data for analyzing strain, which is the next step in earthquake research. There is an ongoing study by the Geodesy Department of KOERI to develop a system which is capable of visualizing strain and velocity maps using continuous data and a processor. The data transfer and processing model of the system is shown in Figure 4. Currently, online data from the KANT continuous station is being processed. After the production of GPS velocities, this data is processed for the strain assessment using the system. Using the data gathered by the campaign-based stations, the system computerizes the strain rates and velocity field maps which are to be the input information for the hazard estimation. However, the temporal resolutions of the data provided by the continuous GPS stations and the campaign-based GPS stations is not the same, and different data collection strategies require separate computation methods. A continuous strain map is estimated from the permanent station data, whereas semiannual or annual strain maps are produced from the campaign-based data, depending on the data collection intervals.

The confluence of the rapidly expanding sensor, computation, and telecommunication industries has allowed for a new instrument concept: the Sensor Web [4]. Sensor Web is a special type of web-centric information system for collecting, modeling, and storing, retrieving, sharing, manipulating, analyzing, and visualizing information of sensors, sensor observations, and associated phenomena [5]. Sensor Web was conceived at the NASA/Jet Propulsion Laboratory in 1997 and the purpose is to extract knowledge from the data. In the future, using sensor web technology, data can be collected from GPS sensor networks and archived, then position information can be produced and used for fully automated visualization of deformation for earthquake research.

## 3. Case Study: Seismic Hazard Assessment by Strain Using Continuous GPS Data

The North Anatolian Fault Zone (NAFZ) is one of the most seismically active faults in the world. Studies monitoring horizontal crustal movements on the western part of NAFZ were started by the Geodesy Department of Kandilli Observatory and the Earthquake Research Institute of Bogazici University in 1990. This region of the country is exposed to a high seismic hazard risk because of the region's tectonics. Three geodetic control networks were established in eastern Marmara (Iznik, Sapanca, and Akyazi regions) in order to monitor crustal displacements. The first period observations had been performed by means of terrestrial methods (theodolite and electromagnetic distance-meter instruments) and these observations had been repeated annually until 1993. Since 1994 GPS measurements have been carried out at the temporary and permanent geodetic control points in the area and the crustal movements have been monitored. In order to investigate tectonic deformation in the Marmara region, GPS campaigns have been performed every year at distributed points that spread over the region by another collaborative project among BU, MIT, TUBITAK, GCM, and ITU. This network includes selected two points from each network mentioned above. Further information about this network can be found in Ergintav *et al.* [2].

In the case study, the crustal strain over the crust of the Marmara region in Turkey has been investigated for seismic hazard assessment using GPS velocity data obtained from these geodetic networks. In order to expand the coverage, MAGNET stations are used in the process. 65 GPS velocities come from MAGNET and also GPS campaigns performed between the years of 2003 and 2005 in Marmara region.

There are different kinds of methods to obtain strain parameters. Geodetic methods of repeated determination of position from GPS sensors are used to obtain and monitor strain accumulation and analysis. Importantly, determination of the small regions in which the strain is assumed as homogeneous affects the results. The method which was developed by Haines and Holt [6] in order to estimate a strain rate and velocity model is followed to carry out this study. A comprehensive overview of the methodology can be found in Haines *et al.* [7]. In this study, the model grid is continuous in longitudinal and latitudinal directions and covers Turkey, between 30°N and 45°S and between 20°W and 45°E. The model is calculated on a regular grid. Each grid area is 0.5° × 0.5° in dimension. Various grid sizes can be examined in future work. Deforming areas are determined according to the seismicity occurrence [8-10]. In total there are 1,500 grid areas, 1,081 of which are the grid areas which cover the deforming regions of Turkey, all other areas are considered to be rigid (Eurasia, African and Arabian plates). The extent of the rigid blocks is also based on the seismicity. Any number of geodetic studies can be combined in this method. In fact, the aim of geodetic measurements by GPS sensors is the determination of position. But they also provide an indirect measure of the rate of seismic productivity of a region. And its spatial derivatives can provide details of tectonic process such as strain.

**Figure 5.**
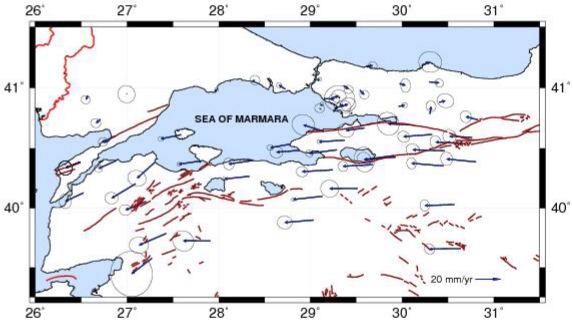
Horizontal velocity field of the Marmara Region in a Eurasia-fixed reference frame (ellipses are at 95% confidence level and the data covers 2003–2005 time intervals).

**Figure 6.**
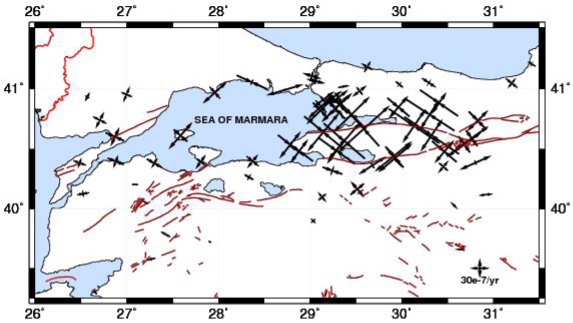
Principal strain rates (ε_1_ and ε_2_) for Marmara region from the inversion of GPS velocities. Inside arrows indicate compression directions and outside arrows indicate extension directions.

According to Haines and Holt [6], three components of strain rate tensor determine a rotation vector function *W*(*x̂*) and it describes the horizontal velocity field u(r) expressed as:

$$u(\hat{x})=rW(\hat{x})\times \hat{x}$$

where r is the radius of the Earth and *x̂* is the position vector on the Earth's surface. The associated horizontal components of strain rate are given by:

$$\begin{array}{c} \varepsilon_{\phi \phi }=\frac{\Theta }{\cos\theta }\cdot \frac{\partial W}{\partial \phi },\\ \varepsilon_{\phi \theta }=\frac{1}{2}(\Theta \cdot \frac{\partial W}{\partial \theta }-\frac{\Phi }{\cos\theta }\cdot \frac{\partial W}{\partial \phi }),\\ \varepsilon_{\theta \theta }=-\Phi .\frac{\partial W}{\partial \theta } \end{array}$$

with *ϕ* and *θ* being longitude and latitude respectively and

$$\begin{array}{c} \Phi =(-\sin\phi ,\cos\phi ,0),\\ \Theta =(-\sin\theta \cos\phi ,-\sin\theta \sin\phi ,\cos\theta ) \end{array}$$

being the unit vectors in the east and north directions. Any region, where W(r) is constant, is considered undeforming (rigid). The Bessel form of a bi-cubic spline interpolation is used to make strain rates continuous at the nodes of the grid. These values are obtained from the least-squares inversion between the observed and predicted values of strain rate and velocity. Figure 5 shows the velocity field representing active deformation rate from the GPS campaigns between 2003 and 2005 in the region. Figure 6 represents the strain rates map derived from this velocity field.

## 4. Results

Results are presented in Tables 1 and 2. They show that the east-west shortening and north-south extension of north-western Turkey are closely related to right-lateral faulting. The velocity value by the evaluation of three GPS campaigns is reaching approximately 25 mm/yr, and the minimum velocity is 0.42 mm/yr. This has an agreement with the region tectonics and the results are very close to the results obtained by Ergintav *et al.* [11]. As it can be seen from the Figure 5, confidence ellipses are larger than velocity vectors at the stations located on the nortern part of the study area. Stations located around south branch of western NAFZ have movements towards west and south-west directions.

Trimble 4000 SSI and 4000 SSE GPS receivers were used to obtain geodetic data. 10-hour/day observation was realized at each campaign-based station. The elevation mask was 10° and the logging interval was 30 seconds. The processing and evaluation of the GPS campaigns was performed with the GAMIT [12] / GLOBK [13] software package. Each campaign were processed using the International Terrestrial Reference Frame; the ITRF2000. Precise orbit by International GPS Service (IGS) was obtained in SP3 (Standard Product 3) format from SOPAC (Scripps Orbit and Permanent Array Center). Earth Rotation Parameters (ERP) came from USNO_bull_b (United States Naval Observatory_bulletin_b). 9-parameter Berne model was used for the effects of the radiation and the pressure. Scherneck model (IERS standards, 1992) was used for the ocean tide loading effect. Zenith Delay unknowns were computed based on the Saastamoinen *a priori* standard troposphere model with 2-hour interval. Iono-free LC (L3) linear combination of L1&L2 carrier phases was used. The model which depended on the height was preferred for the phase centers of the antennas. Loosely-constrained daily solutions obtained from GAMIT were included in the ITRF2000 reference frame by 7 parameters (3 offset-3 rotation-1 scale) transformation with 10 global IGS stations (Table 2).

A present-day deformation field is obtained by GPS velocities in western NAFZ. As it is shown in Figure 6, north-south extension is dominant in Marmara region.

## 5. Discussion

Scientific understanding of earthquakes is vital. Seismic hazard analysis can be defined as the integration of geophysical, geological, and geodetic data to estimate earthquake potential. If the seismic properties of the faults can be analyzed benefiting from data with high spatial and temporal resolution, then earthquake losses can be reduced. As the population increases, urban development and construction work expand on areas susceptible to earthquakes. With a greater understanding of the causes and effects of earthquakes, it may be possible to reduce the damage and loss of life from these destructive phenomena. Continuous and accurate information of relative position is very important for the analysis of crustal deformation and for making long-term earthquake predictions. GPS sensors are tools which can provide this kind of information.

Assessment of strain accumulation throughout Turkey or in specifically targeted areas can be obtained using geodetic data from GPS sensors. Determination of strain accumulation can identify areas of high seismic hazard in Turkey. In order to improve the understanding of the relationship between strain accumulation and seismic hazard assessment, integration of geodetically derived data from regional and national networks with the existing seismic catalog is needed. While GPS measurements indicate elastic deformation for a region, seismicity provides information about the permanent deformation. Seismic data for all Turkey is available, especially since 1970. Furthermore, focal mechanisms are available from both Global CMT catalog [9] and National Earthquake Monitoring Center (NEMC) of KOERI [10]. These data can then be used also to obtain strain and velocity maps.

From the results of this study, we can conclude that the losses of life and property due to earthquake activity in Turkey seem to be unavoidable. However, their magnitude can be reduced. The geodetic deformation field is dominated by right-lateral strike-slip deformation along NAFZ. And western NAFZ where has large urban and industrial centers has the potential for large earthquakes. The eastern NAFZ is also capable of generating major earthquakes in every 3-4 years. To conclude, there is an exigency for a system which will have the ability to computerize earthquake hazard maps as quickly as possible to provide information for making decisions on risk assessments and emergency managements.

## Figures and Tables

**Table 1.** Horizontal GPS velocities of the Marmara Region in a Eurasia-fixed reference frame and 1-sigma uncertainties (plotted with 95% confidence ellipses in Figure 1). RHO is the correlation coefficient between the E (east) and N (north) uncertainties.

**Site**	**Longitude** **(°)**	**Latitude** **(°)**	**E_vel_** **(mm/yr)**	**N_vel_** **(mm/yr)**	**E_sig_** **(mm/yr)**	**N_sig_** **(mm/yr)**	**RHO**
ALAP	31.417	41.201	7.42	5.23	4.35	3.08	-0.076
AKKO	31.198	41.045	7.05	9.77	3.08	2.65	-0.093
CMLN	30.916	40.118	-24.81	2.19	0.97	0.82	-0.063
KDER	30.827	40.735	-9.62	2.49	0.94	0.88	-0.067
TEBA	30.804	40.386	-23.00	2.25	1.07	0.94	-0.052
AGOK	30.761	40.589	-17.73	0.78	1.10	1.05	-0.103
AGUZ	30.680	40.538	-22.79	1.20	0.98	0.88	-0.083
ESKI	30.637	39.658	-24.38	-0.35	0.81	0.81	-0.118
MHGZ	30.570	40.028	-23.34	-0.65	0.91	0.76	-0.048
DGCT	30.462	40.478	-25.01	0.82	0.93	1.08	-0.073
SEYH	30.453	40.351	-25.32	2.15	0.90	0.76	-0.051
CALT	30.405	40.880	4.76	1.19	1.12	1.34	-0.080
SEFI	30.325	40.612	-22.25	-1.63	0.88	0.96	-0.107
KAZI	30.303	40.785	0.79	5.83	0.85	0.79	-0.047
KANR	30.294	41.048	7.88	-0.81	0.66	0.67	-0.094
KFKT	30.229	41.187	5.40	2.48	1.67	1.83	-0.029
SMAS	30.134	40.690	-20.73	1.08	1.83	1.75	-0.091
AKCO	29.973	41.034	4.48	-1.41	1.10	0.78	-0.056
UCG2	29.962	40.846	4.71	0.98	0.40	0.41	-0.038
IUCK	29.929	40.425	-24.64	-1.89	1.99	1.63	-0.062
IGAZ	29.908	40.438	-23.31	-3.09	1.16	1.10	-0.097
DERB	29.681	40.362	-23.49	-1.42	0.82	0.76	-0.067
SILE	29.623	41.179	4.04	0.37	0.63	0.67	-0.063
OLUK	29.585	40.667	-14.29	-1.96	1.10	1.26	-0.061
OVCT	29.539	40.980	3.41	-3.46	0.68	0.75	-0.038
HMZA	29.514	40.164	-22.00	-0.24	1.37	1.42	-0.029
TUBI	29.451	40.787	-1.15	0.80	0.29	0.26	-0.037
DUMT	29.372	40.566	-20.16	-1.16	0.32	0.31	-0.039
KRDM	29.362	41.017	3.79	-2.90	0.67	0.74	-0.057
IBBT	29.321	40.866	2.82	-2.22	1.02	1.18	0.014
HART	29.310	40.927	0.42	-1.60	2.10	2.43	-0.029
KUTE	29.288	40.485	-21.64	-2.77	0.55	0.59	-0.043
KAMT	29.273	40.834	9.78	2.75	1.09	1.23	0.030
ERCT	29.243	40.319	-24.05	-1.44	0.81	0.91	-0.028
YACT	29.238	40.917	4.94	1.14	1.03	1.19	0.000
DRGT	29.145	40.909	8.18	-0.27	0.77	0.85	-0.033
CINA	29.143	40.639	-15.87	5.07	1.66	1.81	-0.091
ULUT	29.131	40.098	-23.02	-2.59	0.30	0.28	-0.041
BAD1	29.118	40.852	0.26	1.21	0.31	0.29	-0.037
YANT	29.113	40.820	-1.05	0.96	0.70	0.76	-0.050
KANT	29.061	41.061	2.90	0.87	0.31	0.30	-0.038
BLOT	29.033	39.899	-22.61	-1.93	1.03	1.19	-0.021
ISTA	29.019	41.104	1.84	0.35	0.29	0.27	-0.037
FIST	28.882	40.481	-18.27	-1.47	0.92	1.01	-0.014
BOZT	28.782	40.534	-15.92	-2.82	0.46	0.48	-0.042
AVCT	28.724	40.989	-4.900	3.10	0.41	0.42	-0.053
YENN	28.373	40.398	-19.10	-2.82	0.93	0.85	-0.069
SELP	28.365	41.052	2.09	1.01	0.75	0.76	-0.051
KART	28.333	40.265	-19.73	-2.10	0.28	0.26	-0.043
MER1	27.962	40.967	1.07	1.88	0.29	0.26	-0.035
BALI	27.906	39.722	-21.20	0.08	1.65	1.69	-0.104
ERDT	27.808	40.393	-17.65	-2.56	0.31	0.30	-0.043
MADT	27.587	40.611	-15.91	-3.29	0.37	0.38	-0.036
ALAN	27.424	39.785	-21.26	-9.15	1.31	1.54	-0.013
KABI	27.301	40.381	-13.88	-12.43	1.28	1.45	-0.106
EGMI	27.269	39.577	-15.95	-12.24	3.61	3.33	-0.020
ASMT	27.204	40.054	-16.01	-6.70	0.87	0.97	-0.046
BKCT	27.091	40.203	-18.83	-11.33	0.93	1.05	-0.037
KRDT	26.999	40.951	-0.44	-0.37	1.39	1.37	-0.051
SEVK	26.880	40.396	-13.90	-6.14	0.61	0.67	-0.029
KVAK	26.871	40.601	-9.59	-4.65	0.69	0.73	-0.004
DOKU	26.706	40.739	-3.45	-3.01	0.71	0.80	-0.048
BGNT	26.570	40.932	-1.56	-2.86	0.66	0.72	-0.037
ATHT	26.524	40.126	-14.28	-6.59	1.17	0.96	-0.042
TYFT	26.487	40.383	-11.54	-4.66	1.12	1.10	-0.050

**Table 2.** Principal strains computed at GPS sites.

**Site**	**Longitude** **(°)**	**Latitude** **(°)**	**ε_1_ (10^-7^ year^-1^)**	**ε_2_ (10^-7^ year^-1^)**	**Azimuth (°)**
ALAP	31.417	41.201	0.20	-0.47	27.579
AKKO	31.198	41.045	0.63	-0.81	39.005
CMLN	30.916	40.118	0.69	-0.03	83.628
KDER	30.827	40.735	1.59	-1.46	45.174
TEBA	30.804	40.386	1.87	-0.22	65.961
AGOK	30.761	40.589	2.30	-1.10	54.433
AGUZ	30.680	40.538	2.35	-1.11	54.292
ESKI	30.637	39.658	0.42	-0.05	92.415
MHGZ	30.570	40.028	0.44	-0.11	135.090
DGCT	30.462	40.478	1.61	-1.86	40.386
SEYH	30.453	40.351	0.80	-0.64	39.668
CALT	30.405	40.880	1.39	-2.67	34.398
SEFI	30.325	40.612	2.16	-3.84	34.155
KAZI	30.303	40.785	1.88	-3.45	32.962
KANR	30.294	41.048	0.26	-0.94	32.693
KFKT	30.229	41.187	0.04	-0.39	120.274
SMAS	30.134	40.690	2.22	-3.52	34.370
AKCO	29.973	41.034	0.22	-0.51	39.173
UCG2	29.962	40.846	1.58	-1.67	40.637
IUCK	29.929	40.425	2.46	-1.44	49.743
IGAZ	29.908	40.438	2.66	-1.44	50.756
DERB	29.681	40.362	1.78	-0.36	57.058
SILE	29.623	41.179	0.69	-0.65	119.909
OLUK	29.585	40.667	3.92	-2.68	48.165
OVCT	29.539	40.980	0.90	-0.53	52.214
HMZA	29.514	40.164	1.06	-0.97	139.743
TUBI	29.451	40.787	2.82	-2.39	45.442
DUMT	29.372	40.566	2.75	-3.14	40.885
KRDM	29.362	41.017	0.58	-0.11	79.503
IBBT	29.321	40.866	1.87	-1.35	46.916
HART	29.310	40.927	12.71	-0.66	51.748
KUTE	29.288	40.485	1.72	-2.61	35.832
KAMT	29.273	40.834	2.10	-1.57	45.480
ERCT	29.243	40.319	0.45	-1.18	19.518
YACT	29.238	40.917	1.33	-0.59	52.017
DRGT	29.145	40.909	1.43	-0.45	52.840
CINA	29.143	40.639	2.76	-2.86	39.432
ULUT	29.131	40.098	0.63	-0.53	140.454
BAD1	29.118	40.852	1.92	-0.91	47.284
YANT	29.113	40.820	2.16	-1.22	45.343
KANT	29.061	41.061	1.00	-0.33	103.166
BLOT	29.033	39.899	0.30	-0.27	134.063
ISTA	29.019	41.104	1.00	-0.52	107.245
FIST	28.882	40.481	1.59	-1.95	36.827
BOZT	28.782	40.534	1.85	-1.78	38.365
AVCT	28.724	40.989	2.09	0.09	73.382
YENN	28.373	40.398	0.89	-0.87	38.472
SELP	28.365	41.052	0.29	-1.92	24.344
KART	28.333	40.265	0.32	-0.56	29.569
MER1	27.962	40.967	1.74	-0.89	48.025
BALI	27.906	39.722	0.07	-0.13	31.147
ERDT	27.808	40.393	0.88	-0.58	35.586
MADT	27.587	40.611	1.94	-1.01	41.376
ALAN	27.424	39.785	0.11	-0.59	29.485
KABI	27.301	40.381	0.53	-0.96	30.775
EGMI	27.269	39.577	-0.15	-0.45	166.474
ASMT	27.204	40.054	-0.07	-0.31	25.166
BKCT	27.091	40.203	0.03	-0.31	1.916
KRDT	26.999	40.951	0.85	-0.62	22.674
SEVK	26.880	40.396	0.66	-0.97	20.530
KVAK	26.871	40.601	1.06	-1.17	24.678
DOKU	26.706	40.739	0.92	-0.83	27.207
BGNT	26.570	40.932	0.49	-0.24	27.546
ATHT	26.524	40.126	0.30	-0.67	169.609
TYFT	26.487	40.383	0.50	-0.75	18.871
